# The role of a pulse-based diet on infertility measures and metabolic syndrome risk: protocol of a randomized clinical trial in women with polycystic ovary syndrome

**DOI:** 10.1186/s40795-017-0142-6

**Published:** 2017-03-07

**Authors:** Laura E. McBreairty, Philip D. Chilibeck, Donna R. Chizen, Roger A. Pierson, Lindsay Tumback, Lauren B. Sherar, Gordon A. Zello

**Affiliations:** 1 College of Pharmacy and Nutrition, University of Saskatchewan, 107 Wiggins Road, Saskatoon, SK S7N 5E5 Canada; 2 College of Kinesiology, Physical Activity Complex, University of Saskatchewan, 87 Campus Drive, Saskatoon, SK S7N 5B2 Canada; 3 Obstetrics, Gynecology and Reproductive Sciences, College of Medicine, 103 Hospital Drive, Saskatoon, SK S7N 0W8 Canada; 4 National Centre for Sport and Exercise Medicine, School of Sport, Exercise and Health Sciences, Loughborough University, Loughborough, UK

**Keywords:** Pulses, Polycystic ovary syndrome, Randomized controlled trial, Metabolic syndrome

## Abstract

**Background:**

Polycystic Ovary Syndrome (PCOS) is an endocrine disorder in women of reproductive age with an estimated prevalence of 5–20% of premenopausal women. The clinical symptoms common to PCOS include menstrual dysfunction, hyperandrogenemia, hirsutism, polycystic ovaries, insulin resistance, and hyperinsulinemia. Women with PCOS are at an increased risk of infertility, obesity and type 2 diabetes mellitus. Insulin resistance and hyperinsulinemia are believed to be key contributing factors to the pathogenesis of PCOS; excessive amounts of insulin are directly associated with the increased ovarian production of androgens and metabolic features of PCOS. Pulse-based diets (e.g., beans, chickpeas) are associated with improved glycemic control and have insulin lowering effects. The purpose of this study is to determine whether a pulse-based diet is more effective than the diet recommended by the National Cholesterol Education Program. The primary outcomes of this study are disease measures related to PCOS, with secondary outcomes including measures related to metabolic syndrome.

**Methods:**

Women with symptoms of PCOS will be recruited for the study and a diagnosis of PCOS will be determined by an obstetrician-gynecologist. Women with PCOS will be randomly assigned to receive either a pulse-based diet or the National Cholesterol Education Program therapeutic lifestyle changes (TLC) diet for 16 weeks while participating in an aerobic exercise program. One hundred participants will be required (drop-out rate of 32%) for recruitment to provide 80% power for detecting a significant difference in fasting glucose (*p* < 0.05). Measures related to infertility, metabolic syndrome, quality of life, dietary intake and physical activity will be assessed pre- and post-intervention with follow up assessment at 6- and 12-months post-intervention.

**Discussion:**

Polycystic ovary syndrome is the most common endocrine disorder in women of reproductive age and there is currently no recommended diet for this population of women. The multidisciplinary nature of this study, including determination of measures related to metabolic syndrome, infertility and physical activity provide a comprehensive assessment of any benefits associated with a pulse-based diet in women with PCOS. The results of this study will help in providing evidence-based recommendations for the optimum diet to reduce symptoms associated with PCOS.

**Trial registration:**

NCT01288638. Trial registered January 13, 2011.

## Background

Polycystic Ovary Syndrome (PCOS) is an endocrine disorder in women of reproductive age with an estimated prevalence of 5%–20% of premenopausal women [1, 2]. The clinical symptoms common to PCOS include menstrual and ovulatory dysfunction, hyperandrogenemia, hirsutism, polycystic ovaries, insulin resistance, and hyperinsulinemia [3]. A diagnosis of PCOS is associated with an increased risk of infertility, dysfunctional bleeding, endometrial carcinoma, obesity, type 2 diabetes mellitus, dyslipidemia, hypertension, and possibly cardiovascular disease [3–6]. The pathogenesis of PCOS is not fully understood; however, insulin resistance and associated hyperinsulinemia that affect 50–70% of women with PCOS are believed to be key contributing factors [7, 8]. Insulin resistance and the subsequent production of excessive amounts of insulin are directly associated with the increased ovarian production of androgens and the features of PCOS [9]. Lifestyle modifications, including diet and exercise, are currently the first-line treatment recommended for women with PCOS [10]; however, there are currently no specific diet recommendations for women with PCOS.

Pulses (e.g., lentils, chick peas, split peas, beans) are high in fiber, have a low-glycemic index, are low in fat, and contain high quality protein. Pulse-based diets are also effective for lowering fasting blood glucose, insulin and blood pressure [11, 12]. These nutritional qualities are important for reducing the risk of metabolic-related and cardiovascular diseases such as diabetes. Pulse consumption has been shown to result in positive effects in individuals at risk for metabolic syndrome [13]. Although clinical trials have demonstrated positive health effects of dietary pulse consumption, some studies have limitations such as insufficient follow up time [11] as well as lack of consistency in dose and disease phenotype [12]. Furthermore, the need for more ‘real world’ trials measuring compliance has been highlighted [11].

In women with PCOS, insulin resistance has been shown to be independent of obesity [14]. Hyperinsulinemia and adiposity exacerbate metabolic and reproductive abnormalities in women with PCOS [15–19]; therefore, our objective is to test the hypothesis that a pulse-based diet can improve symptoms related to metabolic syndrome, infertility and menstrual cycle regularity in women with PCOS. The purpose of this manuscript is to detail the study design and methodology used in a randomized clinical trial involving a diet and exercise intervention in women with PCOS.

The primary outcome measures of this study are disease measures related to PCOS including sex hormones and numbers of ovarian follicles. Secondary outcomes of this study include measures related to metabolic syndrome, quality of life, physical activity and diet.

## Methods

### Design overview

**Fig. 1:**
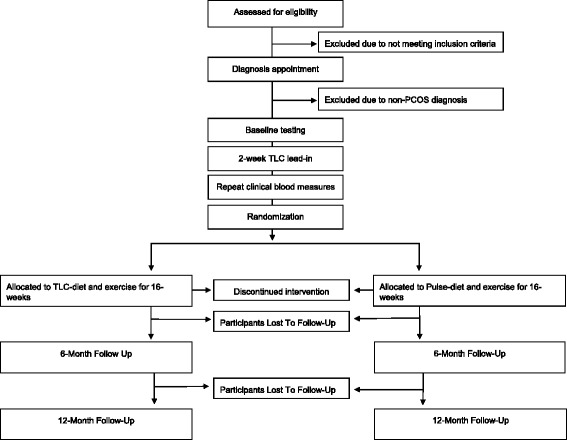
CONSORT flow diagram of study timeline for pulse-based diet intervention in women with polycystic ovary syndrome

The present study is a single-blind, parallel, stratified-randomized clinical trial. The work is funded by Saskatchewan Pulse Growers and Agriculture Agri-Food Canada. The study design is detailed in Fig. 1. The aim of the study is to determine the effect of a pulse-based diet compared to a healthy control diet on disease measures related to PCOS as well as metabolic syndrome risk factors in women with PCOS while they are enrolled in an exercise program. After the study coordinator obtains informed signed consent, study participants will be assessed to determine whether they have PCOS. Women diagnosed with PCOS will undergo baseline testing followed by a 2-week lead-in period on the healthy control diet (therapeutic lifestyle changes (TLC) recommended by the National Cholesterol Education Program). Following the lead-in, participants will repeat baseline blood measures followed by randomization to either the pulse-based diet or the TLC diet for 16-weeks while participating in an aerobic exercise program. Blood measures will be assessed after 9-weeks and at the end of the 16-week intervention, participants will undergo end of intervention testing; follow-up measures will also be assessed at 6-months and 12-months.

The primary outcome of this study is to determine the effectiveness of a pulse-based diet compared to a healthy control diet (TLC diet) for improving PCOS disease measures including sex hormones, menstrual cycle length and, numbers of ovarian follicles. Secondary outcomes include 1) Measures of metabolic syndrome including liver adiposity; 2) Body composition; 3) Quality of life; 4) Physical Activity; and, 5) Diet.

### Setting

The study will be conducted in Saskatoon, SK Canada and is a joint project between investigators in the Colleges of Pharmacy and Nutrition, Kinesiology, and Medicine at the University of Saskatchewan. All medical diagnostic appointments will take place at the Royal University Hospital in Saskatoon. Other assessments will take place at a College of Kinesiology research facility on the University of Saskatchewan campus. Ethics approval and approval for all protocol amendments will be obtained via the University of Saskatchewan Research Ethics Board. All study procedures will be conducted in full compliance with the Tri-Council Policy Statement on the Ethical Conduct for Research Involving Humans, the Declaration of Helsinki on ethical principles for medical research involving humans, and in compliance with the guidelines of the International Conference on Harmonization on Good Clinical Practice.

### Recruitment

Women will be recruited via regular posts on the University of Saskatchewan student and faculty online bulletin board. Women will also be recruited by posters located throughout the University of Saskatchewan, physician offices, health care establishments, and advertisements in local newspapers. The study coordinator will also meet with primary care physicians at clinics in Saskatoon to provide information about the study as well as to distribute consent forms to give to potential participants. Recruitment will also take place via the clinical practice of one of the researchers involved in the study (DRC).

### Population

Eligibility criteria require that volunteers be 18–35-year old females with symptoms of missed or irregular menstrual periods. Participation in the intervention is based on a clinical diagnosis of PCOS. Each participant will be assessed clinically to confirm the diagnosis of PCOS which will be made by an obstetrician-gynecologist specializing in PCOS using criteria specified in the PCOS report of the Androgen Excess-PCOS Society Task Force [3]. Diagnosis of PCOS requires presence of two of the three diagnostic criteria as defined by the Rotterdam consensus: A self-reported history of cycles >35 days in length, hyperandrogenism as defined by a Ferriman and Gallwey score of >6 or hyperandrogenemia [3], as well as polycystic ovaries (PCO), defined as >25 follicles visualized by transvaginal ultrasonography to reflect the newest guidelines for PCO recommended by the Androgen Excess and Polycystic Ovary Syndrome Society [20, 21]. Women with the following conditions will be excluded: Taking anti-seizure or anti-psychotic medications known to induce development of insulin resistance and polycystic ovaries; untreated hyperprolactinemia or thyroid disease; or, excessive adrenal androgen production confirmed by a diagnosis of congenital adrenal hyperplasia, Cushing’s syndrome, or an adrenal tumor. Women using hormonal birth control methods during the prior 3 months or who have a medical condition limiting exercise or consumption of a pulse-based diet (allergies or intolerances) will be excluded. Women will also be excluded from the study if they have an uncontrolled medical condition that interfered with ovarian or systemic hormone production, are pregnant or breastfeeding, or reside outside of the local geographic area.

### Sample size

A pilot study (unpublished) was carried out on preliminary data from 41 women with PCOS randomized to the pulse-based or TLC diet. Thirteen of the 41 women dropped out of the study giving a dropout rate of 32%. Changes in fasting glucose following the intervention (*n* = 28) was used to determine the required sample size for recruitment into the study. Changes in the mean fasting glucose were -0.18 and 0.14 mmol/L for the pulse-diet group and TLC group, respectively, with a population standard deviation of 0.46 and an effect size of -0.70. The pilot data provided 80% power for detecting a significant difference in fasting glucose concentration between groups (*p* < 0.05) following the intervention. It was determined 34 participants per group would be required for the study. Due to the dropout rate of 32%, the recruitment of 100 participants (i.e. 50 per group) was determined necessary.

### Randomization

During the 2-week lead-in period, participants will be randomized to either the pulse based diet or control TLC diet. Participants will be notified of diet allocation via email (and are not blinded). Randomization will be performed by an investigator who is not involved in obtaining or entering participant data. Randomization will be stratified based on the current use of the insulin sensitizing drug Metformin, using a computer-generated allocation schedule with a block size of 4.

### Baseline measures

Prior to starting the 2-week lead-in phase and intervention, all participants will undergo baseline assessments. Assessments include a transvaginal ultrasound, fasting clinical blood measures of reproductive hormones and metabolic syndrome risk factors, glucose tolerance test (GTT) including blood sample for measurement of non-clinical metabolic syndrome risk factors, anthropometric measures, dual-energy X-ray absorptiometry (DXA), physical activity assessment via accelerometer and leisure-time activity questionnaire, 4-day food record, pulse survey questionnaire, and quality of life questionnaire.

### Study phases

The study will consist of 3 phases: 1) Lead-in phase; 2) Intervention; and, 3) Maintenance.

### Lead-in phase

All participants will follow a 2-week lead-in phase with the purpose of controlling for diet variability in baseline blood measures. The lead-in phase involves an initial 1.5-h meeting with a registered dietitian who will discuss the participant’s baseline 4-day food record that will be given during assessment of other baseline measures. The dietitian will provide handouts on and explain the control TLC guidelines to each participant. The guidelines were developed by the National Cholesterol Education Program (NCEP) Expert Panel on Detection, Evaluation, and Treatment of High Blood Cholesterol in Adults [22]. Participants will also be provided with Canada’s Food Guide which gives daily recommendations of 7-8 servings of fruit and vegetables, 6-7 servings of grains, 2 servings of milk or alternatives, and 2 serving of meat or alternatives. The dietitian will explain healthy choices for each food group based on the TLC guidelines which include choosing whole grains and fiber rich foods, lower-fat milk and milk alternatives, and lean meats and meat alternatives, as well as eating less fat and choosing healthy fats more often, and limiting salt, sugar, and alcohol intake.

The lead-in approach has been used by others [23, 24] to control for differences at baseline in measures that might be influenced by previous diet (i.e., ensuring similarity in baseline measures, such as blood parameters, between the 2 groups). After completion of the 2-week lead-in period, participants will repeat the assessment of clinical blood measures of reproductive hormones and metabolic syndrome risk factors.

### Intervention

Following the 2-week lead-in phase, participants will be randomized to either the pulse-based diet or TLC diet and commence the 16-week diet and exercise intervention. The 16-week time period is based on a review of pulse-based diets for improving insulin and glucose levels [11]. In addition, the length of intervention is required to observe changes in outcome measures related to fertility. After 4-weeks of intervention, participants will meet with the study coordinator to discuss any difficulties with participating in the study and hand in the first 4-weeks of study documents which include the daily food and exercise log as well as a 1-day food record and leisure-time exercise questionnaire. After 9 weeks of the intervention, blood will be collected for clinical measures of reproductive hormones and metabolic syndrome risk factors. At the end of the 16-week intervention, baseline measures will be repeated.

### Maintenance

During the final phase of the study, participants will be followed up at 6-months and 12-months post intervention to determine if participants are continuing to follow the “standard of care” recommendations (i.e., TLC diet and exercise) and for volunteers randomized to the pulse-based diet, to determine if they continue to follow a pulse-based diet. Six-month and 12-month follow-up measures include a transvaginal ultrasound, GTT, clinical blood measures of reproductive hormones and metabolic syndrome risk factors, DXA, anthropometric measures, pulse survey questionnaire, and quality of life questionnaire. In addition, physical activity will be assessed via accelerometer at the 6-month follow up.

### Exercise

A gym orientation at the College of Kinesiology research gym facility will be provided to familiarize the participants with the aerobic equipment used during the supervised gym activity. The exercise-training laboratory is fully equipped with aerobic exercise machines and is reserved exclusively for research participants. Aerobic activity will be prescribed as part of the recommended “standard of care” and to reduce variability in this lifestyle factor between participants. Standard of care of exercise training and the TLC guidelines reduces variability among our participants. It is also a design which is ethically sound as it would not be ethical to include a control group that received no care because women with PCOS are a population that requires treatment.

Prior to the beginning of the intervention, the subjects’ current level of physical activity will be assessed via a validated Physical Activity Questionnaire [25]. Exercise training will involve aerobic training 5 days/week for 45 min/day with 3 required sessions in our physical activity facility in the College of Kinesiology at the University of Saskatchewan. This allows research assistants to monitor and support participants as well as address any issues encountered. Exercise will involve walking or training on elliptical machines, rowing machines or stationary bikes, depending on the individual participant’s preferences. Participants will be encouraged to exercise at an intensity of at least 60% of their age-predicted maximal heart rate (i.e., 220 minus age). Instruction will be provided by a research assistant on how to calculate and obtain heart rate measures. This amount of aerobic exercise is the minimal level of exercise shown to be beneficial for improving blood lipids [26].

### Diet

Women in the TLC group will be instructed to continue following the TLC diet prescribed during the lead-in phase. Women in the pulse group will be provided with two pulse meals per day and instructed to follow the TLC guidelines for breakfast and snacks. The pulse-based diet will include a variety of meals prepared with dry peas, lentils, chick peas and faba beans. Two standard meals will be supplied per day to participants (i.e., lunch, dinner) containing approximately 150 g per day of pulses dry weight (250 g per day wet weight). These quantities are based on amounts proven beneficial for lowering blood glucose and lipid levels [13, 27, 28]. The research team will be in contact with participants about food preferences and allergies and participants will have input into which meals are received out of the variety of meals including frozen and fresh options. Meals will be picked up weekly by participants at the exercise facility. Subjects will be counseled to replace foods normally eaten for lunch and supper with the pulse-based food so as not to increase daily caloric intakes.

### Compliance

Compliance to both diet and exercise will be monitored using a daily diet and exercise log book as well as a 1-day food record after every 4-weeks of following the intervention. All participants will be instructed to record daily exercise activity and duration. Women randomized to the pulse group will be required to log which pulse meals are consumed as well as the percentage consumed and both groups will be required to record whether the TLC guidelines are followed each day for all non-prescribed meals, which are all meals for TLC group.

#### Measurements

### Pulse consumption questionnaire

The pulse consumption questionnaire is a modified version of the validated DAILY (Diet Approaches to Increase Lentils in Youth) questionnaire and will be administered at enrollment (prior to TLC guideline counseling) to determine the beliefs and barriers to the consumption of pulses in this population, as well as their current intake of pulses in their diet [29]. The DAILY questionnaire was developed by our research group and has been used in diverse populations.

### Quality of life questionnaire

The quality of life questionnaire, Quality-of-Life Questionnaire for Women with Polycystic Ovary Syndrome, has been previously validated and used to examine the psychological impact of having PCOS and the effect of participation in an intervention [30, 31].

### Accelerometer

Physical activity and time sedentary will be objectively measured using the Actical™ (MiniMitter, Bend, Oregon) accelerometer, a uniaxial accelerometer that detects vertical acceleration. The Actical™ has been validated to measure physical activity in adults [32]. Accelerometers will be used at the beginning of the study to measure the participants’ usual activity (i.e., before exercise program). The Actical (28 x 27 x 10 mm/17 g) will be worn by the participants on a belt worn around the waist with the accelerometer situated at the hip, which will be demonstrated by a research assistant. Participants will be given the monitor on day 1 with data collection programed to record for 7 consecutive days, starting at 12:00 am on day 2 and ending at 12:00 am on day 8. Participants will be encouraged to wear the device on day 1 prior to start of data collection. The participants will be asked to wear the monitor at all times while awake, with the exception of water based activities such as bathing and swimming. The digitized values from the accelerometer are summed over a user-specified interval of 60 s, resulting in a count value per minute (cpm). Data will be downloaded and the raw accelerometer files visually screened for spurious data. Data will be processed using custom KineSoft software (KineSoft, Loughborough, UK). Wear time will be defined by subtracting non-wear time from 24 h. Non-wear time will be defined as at least 60 consecutive minutes of zero counts, with allowance for 1–2 min of counts between 0 and 100. Time spent in different intensities (i.e. sedentary, light, moderate and vigorous) will be calculated using published intensity thresholds for the Actical accelerometer [33, 34], which were used in the Canadian Health Measures Survey [35]. Total time in each intensity will be calculated as the sum of the minutes at a given intensity while the accelerometer is worn.

### Reproductive measures

Ovarian and uterine morphology, including the ovarian volume and the number of ovarian follicles, endometrial thickness and endometrial pattern will be assessed at the Royal University Hospital via transvaginal ultrasound using a GE Voluson® 730 Pro (Zipf, Austria). The ultrasound examination will be timed to occur during days 1-5 of participants’ menstrual cycle, with the first day of menstrual bleeding defined as day 1. Women who do not have cyclical menstrual bleeding will be seen at random times. Levels of estradiol, progesterone and an androgen index (ratio of testosterone to sex hormone binding globulin) will be assessed clinically at the Royal University Hospital and the Saskatchewan Provincial Laboratory.

### Dual-energy X-ray absorptiometry

Fat mass, lean mass, bone mineral content (BMC), bone mineral density (BMD) and hip geometry will be determined using DXA (Hologic© Discovery Wi; Bedford, MA) as previously described [35–39]. All DXA scans will be performed and analyzed by a certified radiology technologist. Whole body BMD and BMC, as well as BMD of the lumbar spine (LS) (L1–L4 vertebrae) and proximal femur regions including the femoral neck, trochanter, intertrochanteric and total hip will also be determined. DXA scans will also be used to determine geometric measures at regions of the hip [40].

### Leisure-time exercise questionnaire

Leisure-time exercise will be determined using a validated questionnaire [25]. The questionnaire will be explained to participants and examples of various levels of exercise will be provided prior to completion.

### 4-Day food record

Participants will be given an initial 4-day food record during baseline measures and will be provided with both oral and written instruction for completion. Participants will be instructed to fill out the record throughout the day as food is consumed and will be instructed on how to provide type, quantity and preparation method. Dietary intake data will be analyzed for energy and nutrient content using the dietary analysis program Food Processor (version 7, ESHA Research, Oregon). This program enables the use of both the USDA Handbook 8 (USDA, 1999) and the Canadian Nutrient File (CNF 1997 version). The database includes more than 20,000 food items, including specialty, ethnic, combination and fast foods. Other food items also can be entered from recipes.

### Glucose tolerance test

A fasted glucose tolerance test will be performed by a physician (DRC) at the Royal University Hospital. An intravenous catheter will be placed in the distal arm with a saline lock attached. Blood samples will be drawn in vacutainer tubes after discarding the saline flush from the IV catheter and the saline flush will be restored between blood sampling times. Following collection of a baseline blood sample, participants will consume a Trutol 75 g Glucose Tolerance Test Beverage (Thermo Fisher Scientific, East Providence, RI) which will be followed by blood collection every 30 min for 2 h. Blood will be collected via 6 ml EDTA or 10 ml empty collection tubes with 3 empty and 2 EDTA tubes used at baseline, 1 empty and 2 EDTA tubes used at 30, 60 and 90 min, and 2 empty and 2 EDTA tubes used at 2 h. Plasma or serum samples will be obtained by hospital staff by aliquoting samples into cryogenic vials which will be frozen at -80 °C for future analysis of insulin and glucose. Plasma samples will also be collected for assessment of other blood measures as described below. At 9-weeks, clinical blood measures will be repeated and blood collected and aliquoted as described above using 1 empty and 2 EDTA tubes. The GTT will correspond with the timing of the ultrasound examination within days 1–5 of participants’ menstrual cycle.

### Measures related to metabolic syndrome

Blood pressure, height, weight and waist circumference will be determined by a research assistant at the College of Kinesiology research facility. Weight will be measured using a calibrated scale and height determined with a standiometer. Waist circumference will be measured after removing clothing from the abdomen, by locating the top of the hip bone (iliac crest) and measuring horizontally at the tip of the landmark. Body mass index (BMI) will be determined as mass (kg) divided by height squared (m^2^). Clinical fasting bloodwork will be completed at the Royal University Hospital and include fasting plasma glucose, insulin, triglycerides, lipoproteins, C-reactive protein, vitamin D and hemoglobin A1C. Fasting bloodwork collected during the GTT or at the 9-week blood collection will be used to determine D-lactate and methylglyoxal by a technician. Liver adiposity will be assessed via ultrasonographic imaging using the ultrasound instrument GE Voluson® 730 Pro (Zipf, Austria) with analysis via Imagyne software to assess for unique echotextural characteristics [41].

### Data management and analysis

The present study will follow intent to treat analysis with measurements and analysis carried out on all participants. All identifying information will be maintained on a secure University of Saskatchewan password protected drive that is shared only with principal investigators and primary research assistants identified on our ethics submission. All hard copies of identifiable information will be securely stored in a locked filing cabinet in a locked room with restricted access. A participant coding system will be used for all data collection which will be stored separately from identifying information.

All raw data obtained will be compiled and reviewed for accuracy on an Excel Spreadsheet. Descriptive statistics will be performed for all outcome measurements for the presentation of group data as means + standard deviation as well as to identify outliers and determine normality of the data.

The treatment effect on the dependent variables will also be assessed by a group by time ANOVA (baseline, 9- and 16-weeks), with repeated measures on the time factor using Statistica (Statsoft, Chicago IL). Any missing data points at 16-weeks will be accounted for by carrying forward the 9-week value. To verify this approach, we will also run a sensitivity analysis including only participants who complete all 3 time points. Any differences determined between the groups at baseline will be used as co-variates in our analysis. Questionnaire data (Quality of Life, Pulse Consumption, Physical Activity) will be presented descriptively. The participants and group categorization will be coded such that the investigators collecting and analyzing data are blinded to which diet the participants were following.

### Monitoring

Monitoring of the study will take place via yearly renewal of ethical approval. The ethics board has the option to review or inspect files at any time. Researchers will have regular contact with participants at the College of Kinesiology research gym facility where any adverse events can be reported. All adverse events will be recorded and University of Saskatchewan guidelines will be followed which require reporting of adverse events to the ethics board if the answer was yes to all 3 of the following: 1). Is the nature of the problem or event unexpected in terms of nature, severity or frequency?; 2). Is the nature of the problem or event related or possibly related (to the intervention)?; and, 3). Does the nature of the problem or event suggest that the research places participants or others at greater risk of harm (including physical, psychological, economic or social harm) than was previously known or recognized, or that were not described in the original application? Due to the short duration of the intervention and low likelihood that either diet will result in any adverse events of concern, there is no data monitoring committee, stopping guidelines or plans for interim analysis.

### Study status

This paper presents an ongoing study which includes follow-up measures that will be carried out. Following completion of the study, results will be presented in peer-reviewed scientific journals with focus on measures of infertility, metabolic syndrome, glucose response, diet, physical activity, and quality of life.

## Discussion

The present study is designed to determine whether a pulse-based diet is equally or more effective than a healthy control diet at reducing symptoms related to metabolic syndrome and reproductive dysfunction in women with PCOS. Although diet intervention studies in women with PCOS have been carried out, this is the first to determine the effects of a pulse-based diet in this population. The study design outlined has several features contributing to the novelty of this research. The diagnoses of PCOS used in dietary intervention studies have primarily used the Rotterdam criteria which defines polycystic ovaries as >12 follicles [42–44]. Recently, it has been suggested that the follicle count recommended by the Rotterdam criteria is too low due to improvements in resolving power of ultrasound images and an accurate diagnosis of PCOS requires a threshold of 26 follicles [21, 45]. The enhanced criterion used in the present study may allow for a more accurate representation of the PCOS population compared to similar intervention studies.

The present study takes a multi-disciplinary approach by incorporating measures from nutrition, physiology, kinesiology, psychology, and reproductive medicine. This approach allows for a comprehensive integration and association of the various components of PCOS which may lead to a better understanding of this multi-faceted syndrome. The inclusion of Kinesiology in the study produces unique data sets such as the use of accelerometers to quantify levels of activity which have been determined via self-reported questionnaires in previous reports [46, 47], 7-day activity records [48], physical activity recall [49] interviews [50], and Pedometer [51]. There is a paucity of physical activity data using accurate and objective measures in women with PCOS [52] and the present study utilizes information obtained using both physical activity questionnaires and accelerometers.

The baseline data collected as necessary for the analyses of the dietary intervention will also serve to characterize women with PCOS. The magnitude of our study with over 100 participants providing baseline measures, will result in an all-inclusive profile of women with PCOS which characterizes their lifestyle (i.e., diet including pulse consumption and exercise), quality of life, various health indicators for risk of disease (e.g., blood parameters, anthropometry, body composition) and infertility.

The primary significance of the research will be to further build on the resources used to develop evidence based dietary recommendations for women with PCOS. Lifestyle is currently the first line of action in treating many symptoms of PCOS and although some diets have been shown to improve specific symptoms of PCOS, there is currently no consensus on an optimal diet for women with the syndrome [53]. We expect to be able to demonstrate whether the insulin lowering effects of a pulse-based diet can better serve women with PCOS to reduce their symptoms.
